# Which Histometric Analysis Approach Is More Reliable for Assessing Histological Bone Tissue Samples?

**DOI:** 10.3390/medicina58101364

**Published:** 2022-09-28

**Authors:** Rodrigo dos Santos Pereira, Carlos Fernando Mourão, Adriano Piattelli, Georgios E. Romanos, Bruno Coelho Mendes, Flavio Giubilato, Pietro Montemezzi, Jadson Júnior Conforte, Geraldo Luiz Griza, João Paulo Bonardi, Eduardo Hochuli-Vieira

**Affiliations:** 1 Department of Oral & Maxillofacial Surgery, University of Grande Rio-UNIGRANRIO, Rio de Janeiro 25071-202, Brazil; 2 Department of Periodontology, Dental Research Administration, Tufts University School of Dental Medicine, Boston, MA 02111, USA; 3 Department of Medical, Oral and Biotechnological Sciences, University “G. D’Annunzio” of Chieti-Pescara, 66100 Chieti, Italy; 4 School of Dental Medicine, Stony Brook University, New York, NY 11794, USA; 5 Department of Oral & Maxillofacial Surgery, Aracatuba School of Dentistry, Sao Paulo State University, Sao Paulo 16066-840, Brazil; 6 Clinical Research Laboratory in Dentistry, Federal Fluminense University, Niteroi 24020-140, Brazil; 7 Department of Dentistry, San Raffaele Hospital, 20132 Milan, Italy; 8 Department of Diagnostic and Surgery, Aracatuba School of Dentistry, Sao Paulo State University, Sao Paulo 16066-840, Brazil

**Keywords:** Bland–Altman analysis, histomorphometric analysis, histological measurement

## Abstract

This study aims to evaluate the grid of Merz and ImageJ methods for histometric quantification, verifying which is more reliable and defining which is most suitable based on the time required to perform. Thirty histological samples of maxillary sinuses grafted with xenografts were evaluated using an optical light microscope attached to an image capture camera and connected to a microcomputer. The images were digitalized and recorded as a TIFF image, and the new bone formation was evaluated using the grid of Merz and ImageJ. The Bland–Altman analysis was used to identify the agreement between the methods and determine suitable future research options. The timing of the quantification was also performed to identify a possible advantage. The mean value for the quantification analysis timing for the grid of Merz was 194.9 ± 72.0 s and for ImageJ was 871.7 ± 264.4, with statistical significance between the groups (*p* = 0.0001). The Bland–Altman analysis demonstrated a concordance between the methods, due to the bias being next to the maximum concordance (−1.25) in addition to the graphic showing the scattering points next to the mean of differences and inside of limits of agreement. Thus, it was demonstrated that the grid of Merz presents reliable outcomes and advantages over the ImageJ methodology regarding the time spent to contour the areas of interest.

## 1. Introduction

Histometric measurements have often been used to assess histological specimens and determine values for the tissues presented. One of the first methods to quantify histological samples was created by Merz, in 1968, and it was named the Merz Hand Grading Scale or grid of Merz. It is a waved grid with 100 countable points that represent 1% of the analysis individually and has been used by numerous researchers since then [1,2,3,4,5,6,7,8,9].

With the advancements of technologies in this area, software tools have been developed to improve the measurement of histometric analysis. For example, ImageJ, a piece of software created by Wayne Rashband, in 1997, is an alternative to the grid of Merz [10,11]. Therefore, the evaluator can measure specific areas demarcating each region with acuity and obtain an exact measure [12].

The grid of Merz allows a subjective evaluation because a greater area is represented by single points, creating doubts on data reliability [4], unlike ImageJ, which means the same area by circling out and measuring it in µm^2^ numerous studies [2,3,4,5,6,7,8,9,10] have been performed using an analogic or digital method to represent histological samples in values. However, the literature did not report which is more reliable.

Martin Bland and Douglas Altman proposed an analysis to determine the limits of agreement between two methods [13]. In their study, it was possible to demonstrate the incorrect use of correlation coefficients and decide if a new method is acceptable. Thus, it is possible to analyze two different methodologies which evaluate the same outcome.

This study aims to determine which method is more reliable between the grid of Merz and ImageJ software using the histometric from matured bone graft as an evaluation sample in the present study. In addition, this study aims to define the most suitable method based on the time required to quantify the areas and a concordance test.

**Hypothesis** **0** **(H0):** *There is no concordance between the methods studied.*

## 2. Materials and Methods

### 2.1. Human Subjects

The present clinical study was performed in Araçatuba Dental School–UNESP, after approval from the ethical committee with number 47711015.4.0000.5420.

### 2.2. Inclusion and Exclusion Criteria

The inclusion criteria were: histological samples with good preservation; histological samples presenting good coloring and sharpness; and histological samples without errors during the histological process. Samples were excluded on the following basis: broken; decolored; samples with errors during the laboratory process and without sharpness.

### 2.3. Number of Samples

A power test was performed based on previous studies [3,4,12] at the website “http://estatistica.bauru.usp.br/calculoamostral” (accessed on 20 February 2021) to determine the number of samples to be evaluated. The test was applied with a mean difference of 16.6, a standard deviation of 9.9, a power of 95%, and a 0.05 significance level in a single tail test. As a result, a minimum of 11 histological samples were required to be reconstructed.

After this, 30 histological samples of maxillary sinuses grafted with Bio-Oss (Geistlich Pharma, Wolhusen, Switzerland) were selected. All specimens were colored with hematoxylin and eosin and then evaluated using an optical light microscope in 12.5× attached to an image capture camera (LeicaR^®^ DC 300F microsystems Ltd., Heerbrugg, Switzerland) connected to a microcomputer. The images were digitalized using the software Axio Vision 4.8 (Carl Zeiss, Oberkochen, Germany) and recorded as TIFF images (tagged image file format).

### 2.4. Evaluation Using the Grid of Merz

**Figure 1:**
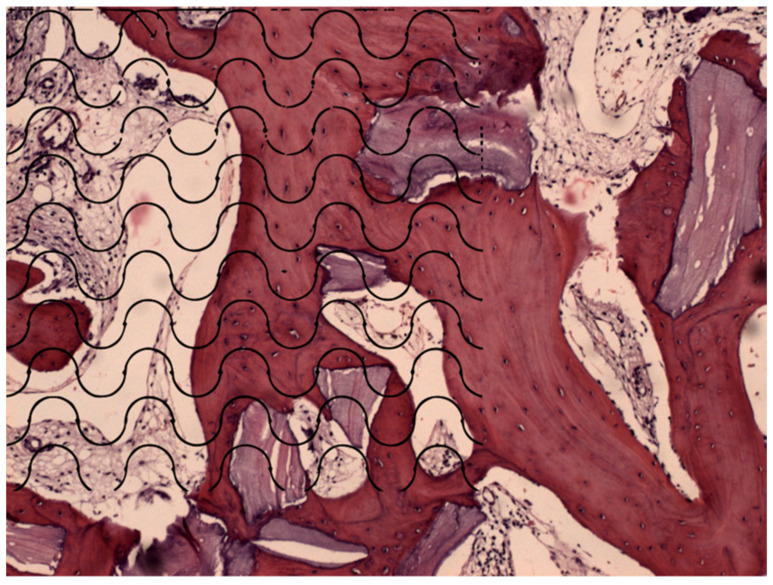
Histological section photography with the grid of Merz attached. Each point represents 1% of the tissue evaluated.

The quantification was performed by one researcher advanced in training. The intraclass correlation coefficient (ICC) was used to determine the evaluator calibration. It was realized as follows: all the 30 samples were quantified in the first measurement. After 30 days, 30% of the samples were quantified again to determine the coefficient. The images were transferred to PowerPoint for Mac (Microsoft, Redmond, WA, USA), and the grid of Merz was attached, overlaying the histological image. Each point represents 1% of the analysis where the new bone is demarcated (Figure 1).

### 2.5. Evaluation Using ImageJ

**Figure 2:**
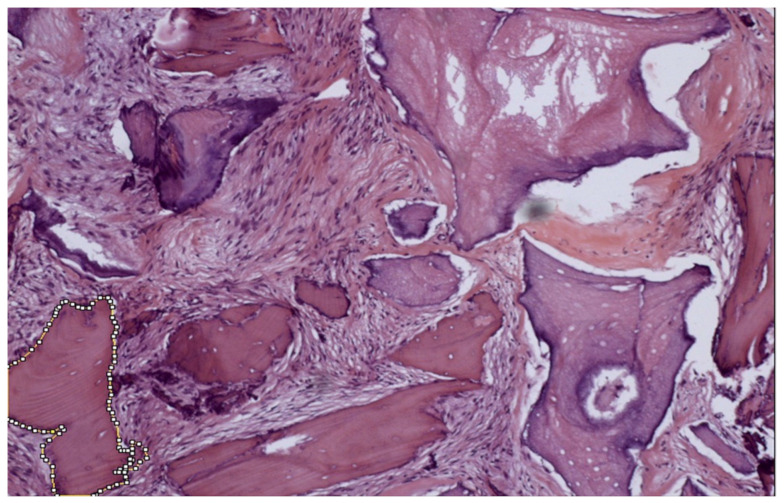
Histological section photography evaluated in ImageJ software.

Another researcher advanced in training performed the quantification using ImageJ 150e (National Institutes of Health, Bethesda, MD, USA), calibrated similarly to the first researcher quantification. The histological images were opened in the software and measured as follows: using the “freehand selections” tool, the referred item to be quantified was contoured, and the tool “measure” was selected, evidencing the area outcomes in µm^2^ (Figure 2). After this, all the results were converted in percentage to be compared with the grid of Merz.

### 2.6. Quantification Analysis Timing

The timing of the quantification analysis was performed by a third assessor who was responsible for conducting both methods to decrease the bias of abilities between the evaluators. In addition, identical calibration of the equipment was ensured. The time was quantified using a digital stopwatch (Vollo, Cotia, São Paulo, Brazil) and the results were expressed in seconds.

### 2.7. Bland–Altman Analysis

The outcomes of both evaluators using the same 30 histological samples were evaluated using the Bland–Altman test (GraphPad Prism 8, San Diego, CA, USA) to identify the concordance of the methods and determine the best option for future research.

The test will inform the mean bias (mean of the results of Grid of Merz and ImageJ) as well as the limits of agreement which will include in the scatterplot. Subsequently, a one-sample *t*-test (GraphPad Prism 8, San Diego, CA, USA) of the differences results was performed to demonstrate a definitive agreement between the methods, which is represented by zero (maximum concordance). For this, if *p* < 0.05, there is no agreement.

### 2.8. Proportion Bias Analysis

Linear regression was performed to determine if the values of the differences between the methods have proportion bias to be only above or only beneath the mean of differences. If *p* < 0.05, the results show this tendency.

### 2.9. Statistical Analysis

The Shapiro–Wilk test was used to verify if the samples had normal distribution for the quantification analysis timing. To compare the timing to quantify the new bone formation of each method, a *t*-test of Students was realized. A priori *p*-value < 0.05 was used.

## 3. Results

### 3.1. Quantification Analysis Timing Results

**Figure 3:**
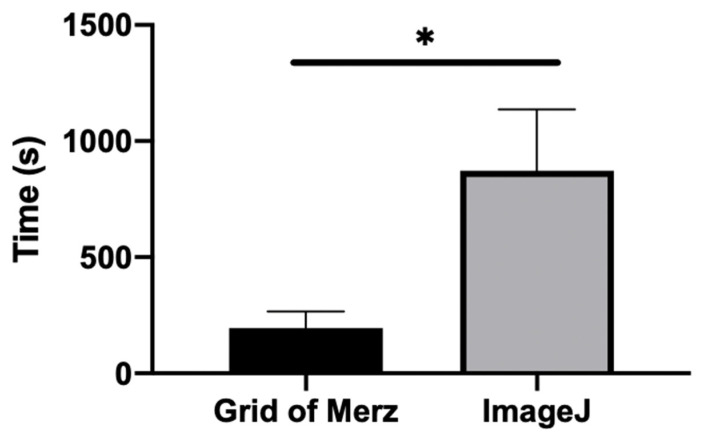
Graphic of the quantification analysis comparing the time (seconds) to quantify the histological samples using the grid of Merz and ImageJ. * *p* < 0.0001.

The mean value for the quantification analysis timing for the grid of Merz was 194.9 ± 72.0 s, and for ImageJ it was 871.7 ± 264.4, with statistical significance between the groups (*p* < 0.0001) (Figure 3).

### 3.2. Result of Analysis of the Differences between Variables

**Figure 4:**
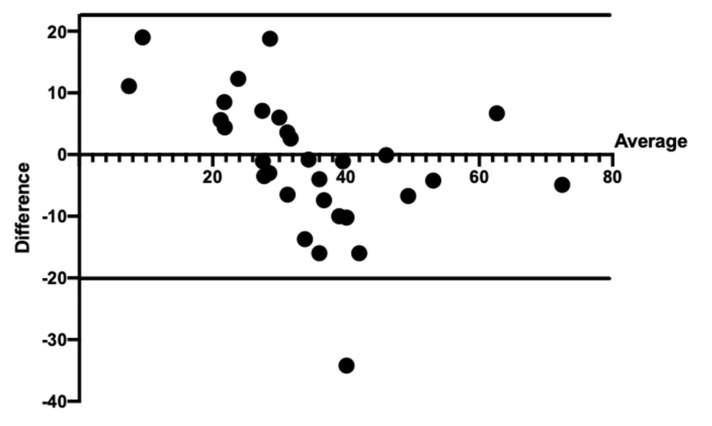
Bland–Altman scatterplot. The red line represents the mean of the differences between the methods. The space between the upper and lower green lines represents the limits of agreement.

The ICC for the evaluator of the grid of Merz was 0.99 and for ImageJ was 0.82, which means an excellent correlation according to Cicchetti and Domenic [14]. The mean value of new bone formed for the grid of Merz was 33.7 ± 12.2% and 35.0 ± 16.8% for the ImageJ evaluation. The bias of the differences between the methods was −1.25; the upper limit of limits of agreement (LA) was 22.8, and the lower limit was 20.3. The *t*-test evidenced a concordance between the methods with *p* = 0.53 (Table S1). The graphic shown a suitable agreement due to the major scattering points relatively close to the mean bias line and inside of the LA, which means reliability between the methods (Figure 4). Thus, the null hypothesis (H0) was accepted.

The linear regression demonstrated a non-normal distribution (*p* = 0.01), which indicates a proportion bias and heterogeneous distribution. Thus, the values of the methods tend to error just above or beneath the average line.

## 4. Discussion

The results of the present study showed the differences between the grid of Merz and ImageJ software using mature bone (after six months of the healing process) from sinus lift procedures as samples for evaluation. Thus, the current method can be applied to other tissues assessment (e.g., bone, connective tissue). Therefore, the analysis included in this research does not directly relate to the maxillary sinus bone augmentation procedure but the method of tissue evaluation.

For many years, the correlation coefficient was used to indicate agreement between two measurements. However, this analysis could not report the strength of this relationship due to the use of variables, not differences [15,16]. The Bland–Altman analysis can be described using a scatterplot graphic representing information about the agreement between the methods studied. The closer the scatter points are to the mean bias line, the more in agreement both methods are [17]. The scatterplot of the present study showed dispersed points. Nevertheless, more than 95% of the differences between the grid of Merz and ImageJ were within of limits of the agreement. Thus, both methods are reliable.

The idealization of this study was to identify the most suitable and reliable manner to measure histology samples, using the analogic or digital method. The use of software implies the best accuracy, due to the possibility of handling the total area of a specific part of the samples, as purposed by ImageJ. On the contrary, the use of the grid of Merz allows the measure of particular points, which can lead to doubts about the results obtained. In the present study, the outcomes demonstrated that using the Bland–Altman analysis and the timing quantification, the grid of Merz is a suitable method and is faster to perform than ImageJ. In the graphic, it is possible to observe that the scatting points are in the range of both means methods as well as inside of the LA. Thus, there is concordance between the grid of Merz and Image J.

The purpose of using two evaluators for this agreement evaluation was to strengthen the reliability of the study. The individual analysis in each method prevents biased interpretation and simulates the reality because previous histometric studies used only one of them [4,12]. Another researcher realized the quantification analysis timing to decrease the skill bias between the other two evaluators. The time of the histometric analysis using the ImageJ was higher than using the grid of Merz. These outcomes suggest that in studies with many samples, the difference in time to conduct the quantification is advantageous compared to those using fewer samples.

The method with higher reliability is more informative and reliable in terms of standards and recommendations. Further studies are recommended to expand the study sample of focus group members, which may improve the reliability of the evaluation methods and the outcome. The Bland–Altman analysis was used to identify the reliability of the evaluations using the grid Merz or ImageJ due to its higher precision. Another statistical test, which can be suggested, is Pearson’s correlation coefficient. However, the results could demonstrate a high correlation but with less reliability. On the other hand, the present study informed how concordant both methods were regardless of the coefficient degree. In the present study, the outcome showed concordance for both methods evaluated, answering this study’s purpose. Thus, the null hypothesis was accepted.

The limitations to using the Bland–Altman analysis are the requirement of a normal distribution and an adequate sample size [17]. Another question is about the methods used in the present study. Recent methods have been used to quantify histological samples, even in three-dimensional studies [18]. However, it is impossible to evaluate the morphology of the cells.

## 5. Conclusions

In conclusion, both methods can be used to measure histological samples, due to the concordance and reliable results of the Bland–Altman analysis. However, the grid of Merz presented an advantage regarding the time spent required, compared with ImageJ being a better indicator for studies with many samples.

## Data Availability

Not applicable.

## Supplementary Materials

The following supporting information can be downloaded at: https://www.mdpi.com/article/10.3390/medicina58101364/s1, Table S1: Data for the new bone formation using the grid of Merz and Image J.
