# Supplementary material for: Which Histometric Analysis Approach Is More Reliable for Assessing Histological Bone Tissue Samples?
Source: Medicina (Kaunas). 2022 Sep 28;58(10):1364. doi: 10.3390/medicina58101364 (PMC9611878; doi:10.3390/medicina58101364)
Supplement: Supplementary file 1 [file medicina-58-01364-s001.zip › medicina-1935750-supplementary.pdf]

Table S1. Data for the new bone formation using the grid of Merz and ImageJ

Samples	Mean of bone formed using the grid of Merz (%)	Mean of bone formed using ImageJ (%)	Difference between methods	Mean of methods
1	46	46,1	-0,1	46,05
2	33	29,4	3,6	31,2
3	39	40,1	-1,1	39,55
4	27	40,7	-13,7	33,85
5	24	19,6	4,4	21,8
6	33	40,4	-7,4	36,7
7	26	29,5	-3,5	27,75
8	27	28,1	-1,1	27,55
9	27	30	-3	28,5
10	38	19,2	18,8	28,6
11	26	17,5	8,5	21,75
12	34	50	-16	42
13	28	44	-16	36
14	35	45,2	-10,2	40,1
15	34	38	-4	36
16	34	34,8	-0,8	34,4
17	33	27	6	30
18	13	1,9	11,1	7,45
19	33	30,4	2,6	31,7
20	30	17,7	12,3	23,85
21	28	34,5	-6,5	31,25
22	19	0	19	9,5
23	31	23,9	7,1	27,45
24	34	44	-10	39
25	51	55,2	-4,2	53,1
26	23	57,2	-34,2	40,1
27	66	59,3	6,7	62,65
28	24	18,4	5,6	21,2
29	46	52,7	-6,7	49,35
30	70	74,9	-4,9	72,45
Mean of bone formed ± SD	33.7 ± 12.2	35.0 ± 16.8		
Average of differences			-1,3	
SD of differences			11,0	
t-test			0.608	
